# Separate Ionotropic and Metabotropic Glutamate Receptor Functions in Depotentiation vs. LTP: A Distinct Role for Group1 mGluR Subtypes and NMDARs

**DOI:** 10.3389/fncel.2016.00252

**Published:** 2016-11-07

**Authors:** Amira Latif-Hernandez, Enrico Faldini, Tariq Ahmed, Detlef Balschun

**Affiliations:** Laboratory of Biological Psychology, KU LeuvenLeuven, Belgium

**Keywords:** depotentiation, metabotropic glutamate receptors, metabotropic NMDA receptor function, long-term potentiation, mouse hippocampus

## Abstract

Depotentiation (DP) is a mechanism by which synapses that have recently undergone long-term potentiation (LTP) can reverse their synaptic strengthening within a short time-window after LTP induction. Group 1 metabotropic glutamate receptors (mGluRs) were shown to be involved in different forms of LTP and long-term depression (LTD), but little is known about their roles in DP. Here, we generated DP by applying low-frequency stimulation (LFS) at 5 Hz after LTP had been induced by a single train of theta-burst-stimulation (TBS). While application of LFS for 2 min (DP2′) generated only a short-lasting DP that was independent of the activation of *N*-methyl-D-aspartate receptors (NMDARs) and group 1 mGluRs, LFS given for 8 min (DP8′) induced a robust DP that was maintained for at least 2 h. This strong form of DP was contingent on NMDAR activation. Interestingly, DP8′ appears to include a metabotropic NMDAR function because it was blocked by the competitive NMDAR antagonist D-AP5 but not by the use-dependent inhibitor MK-801 or high Mg^2+^. Furthermore, DP8′ was enhanced by application of the mGluR1 antagonist (YM 298198, 1 μM). The mGluR5 antagonist 2-Methyl-6(phenylethynyl) pyridine (MPEP, 40 μM), in contrast, failed to affect it. The induction of LTP, in turn, was NMDAR dependent (as tested with D-AP5), and blocked by MPEP but not by YM 298198. These results indicate a functional dissociation of mGluR1 and mGluR5 in two related and consecutively induced types of NMDAR-dependent synaptic plasticity (LTP → DP) with far-reaching consequences for their role in plasticity and learning under normal and pathological conditions.

## Introduction

Neurons have the ability to modify their architecture and functionality in response to recent activity, which ultimately serves for learning (Abraham and Bear, 1996). For example, natural and artificial patterns of afferent activation have been demonstrated to induce persistent forms of synaptic plasticity such as long-term potentiation (LTP), an activity-dependent increase of synaptic efficacy, and long-term depression (LTD), a reduction in synaptic strength (Bliss and Collingridge, 1993; Citri and Malenka, 2008; Collingridge et al., 2010). *N*-methyl-D-aspartate receptor (NMDAR)-dependent forms of LTP and LTD are prominent in the hippocampus (HC) and other brain regions and were shown to occur during learning and memory formation (Whitlock et al., 2006; Abraham, 2008; Dong et al., 2013), The most widely explored experimental system for studying the molecular basis of memory has been NMDAR-dependent LTP in the CA1 region of HC (Bliss and Collingridge, 1993; Citri and Malenka, 2008; Mayford et al., 2012). In contrast, another form of synaptic plasticity, depotentiation (DP), first described by Hesse and Teyler (1976), has received far less attention. DP is the partial or complete reversal of LTP and is usually induced by long trains of low-frequency stimulation (LFS) given within a brief time window after LTP induction (Barrionuevo et al., 1980; Fujii et al., 1991; O’Dell and Kandel, 1994; Stäubli and Chun, 1996; Balschun et al., 1999b). The less time has passed after LTP induction, the easier DP can be induced (i.e., shorter LFS trains can be used for induction), and the more pronounced DP is expressed. Thus, the inducibility of DP decreases and is counteracted by the progressing consolidation of LTP after induction (Fujii et al., 1991; O’Dell and Kandel, 1994; Stäubli and Chun, 1996; Lynch and Gall, 2013).

Although DP shows a number of similarities to homosynaptic LTD such as input-specificity, inducibility by LFS and the dependence on the activation of protein phosphatases (O’Dell and Kandel, 1994), there is also clear evidence that DP and homosynaptic LTD are two distinct phenomena. For instance, the following criteria apply only to DP but not homosynaptic LTD: (i) DP cannot be induced at naïve synapses, i.e., without previous induction of LTP; (ii) it can be evoked at all ages *in vitro* (Wagner and Alger, 1995; Milner et al., 2004); (iii) it can be induced by LFS at 5 Hz, a frequency which neither generates LTD nor LTP because it is in the transition zone of frequency-response curves for the induction of synaptic plasticity (Bienenstock et al., 1982); (iv) it depends critically on the activation of adenosine A1 receptors (Larson et al., 1993; Huang et al., 1999; Dias et al., 2013); and (v) it is not blocked by application of the GABA_B_ receptor antagonist CGP 35348 (Wagner and Alger, 1995).

While recent research has advanced our understanding of the mechanisms responsible for DP, many issues remain unsolved or controversial. In particular, there is no agreement on whether NMDA or metabotropic glutamate receptors (mGluRs) must be engaged for DP induction (reviewed by Huang and Hsu, 2001; Sanderson, 2012). Previously, it was reported that the induction of DP by low-frequency-stimulation is NMDAR mediated (Fujii et al., 1991; O’Dell and Kandel, 1994; Huang et al., 2001). However, others could not replicate these findings and identified mGluRs instead as being necessary for DP induction (Bashir and Collingridge, 1994; Bortolotto et al., 1994). mGluRs are G-protein-coupled receptors (GPCRs) that are divided into three groups (I, II, III) based on their sequence homology, transduction mechanisms and pharmacology (Conn and Pin, 1997). Activation of group I mGluRs (mGluR1 and 5) leads to the hydrolysis of phosphatidylinositol 4,5-bisphosphate into inositol 1,4,5-triphosphate (IP3) and diacylglycerol (DAG), both required for intracellular Ca^2+^ release and activation of protein kinase C (PKC), respectively (Nakanishi, 1994). Experimental evidence indicates that both subtypes of group I mGluRs have distinct functions in synaptic plasticity. For example, the functional importance of mGluR1 and mGluR5 in the induction and maintenance of hippocampal CA1-LTP, respectively, seems to be contingent upon the type of potentiation induced (e.g., weak vs. strong), the dendritic subregion and the species investigated (Bortolotto et al., 1994; Wilsch et al., 1998; Balschun et al., 1999a; Raymond and Redman, 2002; Nagaraja et al., 2005; Neyman and Manahan-Vaughan, 2008). A useful pharmacological tool to explore the function of mGluR1 and mGluR5 in the nervous system is using potent selective, non-competitive antagonists (Gasparini et al., 1999; Knöpfel, 2007).

The role of group I mGluRs in LTD has been mostly investigated by chemically induced types of LTD, where application of the group I mGluR agonist DHPG [(S)-3,5-dihydroxyphenylglycine] was found to cause a pronounced and lasting depression (Schnabel et al., 1999; Huber et al., 2000; Collingridge et al., 2010). In agreement with the notion that DP and LTD share certain induction mechanisms (Huang et al., 2001), application of DHPG briefly after LTP-induction results in DP (Zho et al., 2002; Delgado and O’Dell, 2005; and unpublished laboratory findings).

Interestingly, activation of group I mGluRs in hippocampal neurons leads to potentiation of NMDA-mediated responses (Conn and Pin, 1997), indicating an interaction between group I mGluR and NMDAR dependent mechanisms. Consistent with these findings, activation of NMDARs potentiates mGluR5 responses via activation of the serine/threonine protein phosphatase calcineurin (PP2B) which dephosphorylates mGluR5 (Alagarsamy et al., 1999, 2005). A recent study described an involvement of mGluR5 in LTP by gating NMDAR-dependent LTP (Kwag and Paulsen, 2012). Therefore, although mGluRs are accepted to be important for various forms of synaptic plasticity (Bortolotto et al., 2005), their specific role in DP was not addressed in detail yet, and in particular, it is not known whether NMDARs and mGluRs interact to produce DP.

To pharmacologically characterize the role of group I mGluR- and NMDAR-dependent mechanisms in DP in the CA1-region of adult mice, we employed a new protocol for DP induction in the Schaffer collateral-commissural pathway and examined the effect of selective antagonists of mGluR1, 5 and NMDAR, respectively, when applied during different phases of the protocol. We confirm that the induction of LTP by theta-burst-stimulation (TBS) and DP by LFS (5 Hz) requires the activation of NMDARs. Interestingly, our data support a metabotropic function of NMDARs during DP-induction. Further, we find that the group I mGluR subtypes are differentially involved in both types of synaptic plasticity. DP is “tonically” suppressed by mGluR1 activity but not affected by mGluR5. TBS-LTP, in contrast, is contingent on the activation of mGluR5, but not of mGluR1. Our findings demonstrate a functional dissociation of mGluR1 and mGluR5 in two related types of NMDAR-dependent synaptic plasticity with implications for their role in plasticity and learning under normal and pathological conditions.

## Materials and Methods

### Slice Preparation

Mice (8–12 weeks old) were killed by cervical dislocation in accordance with KUL Institutional, State and Government regulations, and HC was rapidly dissected out into ice-cold (4°C) artificial cerebrospinal fluid (ACSF), saturated with carbogen (95% O_2_/5% CO_2_). ACSF consisted of (in mM): 124 NaCl, 4.9 KCl, 24.6 NaHCO_3_, 1.20 KH_2_PO_4_, 2.0 CaCl_2_, 2.0 MgSO_4_, 10.0 glucose, pH 7.4. Transverse hippocampal slices (400 μm thick) were prepared from the dorsal area of the right HC with a tissue chopper and placed into a submerged-type chamber, where they were kept at 32°C and continuously perfused with ACSF at a flow-rate of 2.4 ml/min. After 90 min incubation, a bipolar tungsten electrode was placed in CA1 *stratum radiatum* for stimulation and a glass electrode (filled with ACSF, 3–7 MΩ) about 200 μm apart for recording of field excitatory postsynaptic potentials (fEPSPs). Signals were amplified by a differential AC Amplifier Model 1700 (A-M Systems), fed through a Power1401 data acquisition interface (Cambridge Electronic Design Limited) and analyzed by custom-made software. The time course of the fEPSP was calculated as the descending slope function for all experiments. After input/output curves (I/O) had been established, the stimulation strength was adjusted to elicit a fEPSP-slope of 35% of the maximum and kept constant throughout the experiment. During baseline recording, three single responses were evoked at a 10 s interval by biphasic stimulation (0.1 ms pulse width) and averaged and these measurements were repeated.

### LTP Induction Protocol

To induce a weak, unsaturated form of LTP, a single TBS was employed, consisting of 10 burst of four stimuli at 100 Hz separated by 200 ms (double pulse width) followed by recording of evoked responses at 1, 4, 7 and 10 min post LTP-induction. Thereafter, recording was continued every 5 min until the end of experiments.

### DP Induction Protocol

Six minute after the induction of TBS-LTP, either a weak DP was induced by LFS at 5 Hz for 2 min (DP2; Balschun et al., 1999b), or a more robust DP was generated by LFS at 5 Hz for 8 min (DP8). Subsequently, evoked responses were recorded 1, 4 and 7 min after completion of LFS, and thereafter every 5 min until the end of experiments.

### Drug Application

All drugs were obtained from Tocris or Abcam and stored as stock solutions at −20°C until the day of the experiment, at which they were dissolved to the desired final concentration in ACSF and applied via the perfusion line. For LTP experiments, all drugs were applied 6 min prior to the TBS train for a total of 30 min. For DP experiments, all drugs were applied from 6 min prior to the LFS train (i.e., immediately after LTP induction) for a total of 30 min. To distinguish between functional effects caused by activation of either mGluR1 or mGluR5 during LTP and DP, we used the high-affinity, selective, and non-competitive mGluR1 antagonist YM298198 (YM, 6-Amino-*N*-cyclohexyl-3-methylthiazolo[3,2-*a*]benzimidazole-2-carboxamide hydrochloride; 1 μM; Kohara et al., 2005) with an IC_50_ of 24 nM in physiological assays (Fukunaga et al., 2007; Knöpfel, 2007) and the potent, highly selective, non-competitive antagonist at the mGlu5 receptor subtype 2-Methyl-6 (phenylethynyl) pyridine (MPEP) hydrochloride, 40 μM (IC50 = 36 nM; Gasparini et al., 1999), respectively. The involvement of NMDAR in the induction of LTP and DP was tested with the widely used competitive NMDAR antagonist (2*R*)-amino-5-phosphonovaleric acid (D AP5; 50 μM). A putative metabotropic function of NMDARs was tested with the use-dependent open channel blocker MK-801 (dizocilpine; Huettner and Bean, 1988) or by increasing the concentration of 2.0 MgSO_4_ in ACSF to 10 mM which prevents the opening of the channel pore of the NMDAR (Collingridge and Lester, 1989). All drug experiments were interleaved with vehicle controls.

### Statistical Analysis

All data are presented as mean ± SEM, where “n” refers to the number of animals tested. Differences between mean values (time series) were examined using two-way analysis of variance with repeated measures (RM-ANOVA) with Fisher’s least significant difference (LSD) test for *post hoc* comparison. All other group comparisons were done with two-tailed Student *t*-test or one-way ANOVA. Differences with *p* ≤ 0.05 were considered significant.

## Results

### Application of the mGluR5 Antagonist MPEP Prevents the Induction of LTP

**Figure 1:**
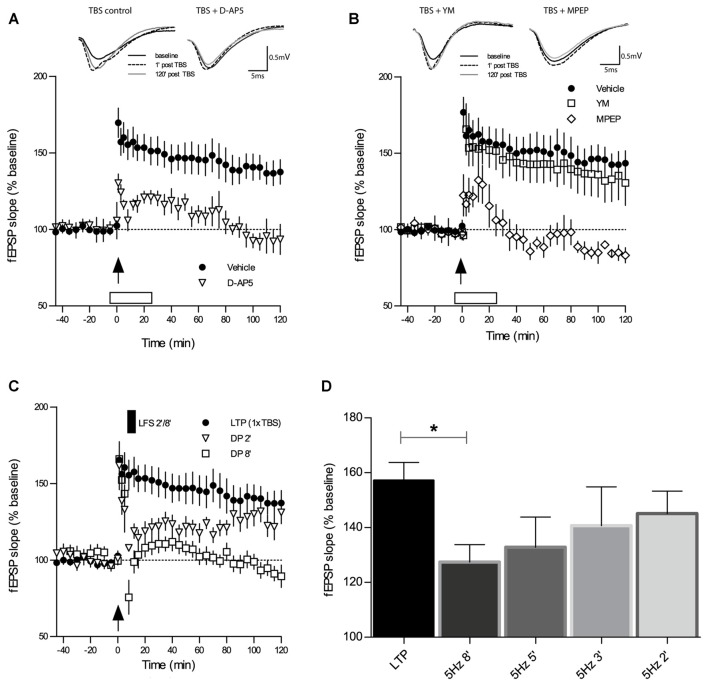
**Theta-burst-stimulation (TBS)-induced long-term potentiation (LTP) is sensitive to inhibition of *N*-methyl-D-aspartate receptors (NMDAR) and metabotropic glutamate receptor 5 (mGluR5) but not of mGluR1. (A)** The selective NMDAR antagonist D-AP5 (50 μM) causes a marked impairment of LTP (*n* = 7) induced by a single TBS (indicated by an arrow) when compared to the vehicle group of slices (*n* = 13). Error bars represent SEM, *p* = 0.0285. **(B)** Inhibitors of mGluR1 and mGluR5 have contrasting effects on TBS-LTP. Whereas the mGluR1 inhibitor YM 298198 (1 μM) does not affect TBS-LTP (*n* = 6), the GluR5 inhibitor 2-Methyl-6(phenylethynyl) pyridine (MPEP; 40 μM) causes a profound blockade of TBS-LTP (*n* = 6) compared to vehicle (*n* = 13; same group of slices for vehicle application as in 1A). Error bars represent SEM, *p* < 0.0001. Traces show representative examples of field excitatory postsynaptic potentials (fEPSPs) recorded during baseline (black line), 1 min post-TBS induction (broken line) and 120 min post-TBS (gray line). Calibration bars: 0.5 mV and 5 ms. Open box indicates time and duration of drug application for 30 min, from 6 min before tetanization until 24 min thereafter. **(C)** Depotentiation (DP) induced by a single TBS (1×TBS) followed by low-frequency stimulation (LFS) at 5 Hz for 2 min (DP2) evoked only a short-lasting depression of fEPSPs that was not significantly different from LTP controls (main effect of group: *F*_(1,19)_ = 2.77, *p* = 0.1122; RM-ANOVA on 120 min post-LFS). In contrast, LFS for 8 min (DP8) caused a significant DP (*F*_(1,20)_ = 17.13, *p* = 0.0005; RM-ANOVA). **(D)** The magnitude of *in vitro* DP depends on the duration of LFS (5 Hz). By varying the duration of LFS (always applied from 6 min after LTP induction), an “LFS duration-response curve” was observed wherein longer trains resulted in stronger DP (control LTP, *n* = 10; LFS 2 min (DP2), *n* = 8; LFS 3 min, *n* = 7; LFS 5 min, *n* = 7; LFS 8 min (DP8), *n* = 8). Bar graphs represent the remaining percentage of potentiation measured in each conditioning group 90 min after TBS application. Note that DP8 protocol was the most effective in generating a robust DP when compared to control LTP. Error bars represent SEM, *p* = 0.0206; *p ≤ 0.05.

Types of synaptic plasticity that follow each other at close intervals like LTP and DP are supposedly intimately linked at the molecular and functional level. Thus, certain properties of LTP will most likely affect specific properties of DP. For this reason, we first characterized TBS-LTP under our experimental conditions with regard to the importance of NMDA and group I mGluR receptor activation. As depicted in Figure 1A, application of the broad-spectrum NMDAR antagonist D-AP5 (50 μM) 6 min prior to the induction of LTP by 1×TBS led to a reduction in both, the induction and maintenance of LTP and only a short-lasting potentiation was obtained (TBS + D-AP5: 1 min 130 ± 6%, 120 min 93 ± 10%, *n* = 7; control TBS: 1 min 176 ± 10%, 120 min 144 ± 9%, *n* = 11; main effect of group for 2 h post induction: *F*_(1,13)_ = 7.937, *p* = 0.0145, RM-ANOVA).

To investigate the requirement for distinct group I mGluR subtypes during TBS-LTP, we selectively bath-applied mGluR1 and mGluR5 antagonists. Previous studies have reported that activation of mGluR1 initiates a signaling cascade that involves stimulation of PKC and ultimately potentiation of NMDARs (Zukin et al., 1997; Skeberdis et al., 2001; Kwag and Paulsen, 2012). Surprisingly, our experiments revealed that TBS-LTP was not affected by the mGluR1 antagonist YM (1 μM; Figure 1B; TBS + YM: 1 min 166 ± 17%, 120 min 131 ± 15%, *n* = 6; control TBS: 1 min 176 ± 10%, 120 min 144 ± 9%, *n* = 11; main effect of group for 2 h post induction: *F*_(1,17)_ = 0.11, *p* = 0.7419, RM-ANOVA).

In contrast, the mGluR5 antagonist MPEP (40 μM) caused a significant blockade of LTP induction and maintenance (Figure 1B; TBS + MPEP: 1 min 122 ± 14%, 120 min 83 ± 5%, *n* = 6; control TBS: 1 min 176 ± 10%, 120 min 144 ± 9%, *n* = 11; *F*_(1,10)_ = 70.46, *p* < 0.0001, RM-ANOVA). These findings demonstrate that activation of mGluR5 but not mGluR1 is an essential requirement for the induction of weak TBS-LTP and its maintenance.

LFS was documented to induce DP in rat hippocampal slices (CA1) that had been subjected to TBS-induced LTP (Larson et al., 1993; Stäubli and Chun, 1996). We proceeded to replicate these findings in slices of adult mice and to establish a robust protocol for our studies on DP. First, we tested a DP induction protocol which consisted of a single TBS (1×TBS) followed by LFS at 5 Hz for 2 min (DP2). DP2 had proven its efficacy in reversing TBS-LTP in a previous study in the dentate gyrus (Balschun et al., 1999b). As shown in Figure 1C, in our study DP2 evoked a short-lasting depression of fEPSPs that was significantly different from LTP controls (not depicted) for 30 min after LFS delivery (main effect of group: *F*_(1,19)_ = 11.41, *p* = 0, 0032; two-way RM-ANOVA on 30 min recording interval post-LFS). However, this weak form of DP did not last, and when the entire recording interval is considered, no statistical significant difference was detected (not depicted; main effect of group: *F*_(1,19)_ = 2.774, *p* = 0.1122; two-way RM-ANOVA on 120 min post-LFS).

### The Magnitude of DP in Slices of Adult Mice Depends on the Duration of LFS

Because of the unexpected failure of DP2 to induce long-lasting DP in CA1 we re-tested DP2 interleaved with stronger protocols, which consisted of episodes of 3, 5 and 8 min of 5 Hz LFS, always starting from 6 min after LTP induction (1×TBS), in the same way DP2 was earlier tested. In these experiments we found that the effectiveness of inducing DP was proportional to the duration of LFS. When the amount of the remaining potentiation was measured in each conditioning group 90 min after LTP induction, we found that an LFS episode of 5 Hz for 8 min (DP8) was the most effective in generating a robust DP (Figure 1D; mean fEPSP % change at 90 min post-TBS control LTP: 157 ± 6%, *n* = 10; 5 Hz 2 min (DP2): 145 ± 8%, *n* = 8, *p* = 0.2639; 5 Hz 3 min: 140 ± 14%, *n* = 7, *p* = 0.2621 unpaired *t*-tests vs. control LTP; 5 Hz 5 min: 133 ± 11%, *n* = 7, *p* = 0.0624; 5 Hz 8 min (DP8′): 127 ± 6%, *n* = 8, *p* = 0.0206). Thus, DP8 proved to be a robust electrical protocol to reliably induce DP and was used, therefore, to examine the cellular mechanisms underlying DP in the following experiments (See Figure 1C for the time course of DP8).

### mGluR1 and NMDAR are Reciprocally Responsible for DP Induced by 8 min LFS and Work Independently of Each Other

**Figure 2:**
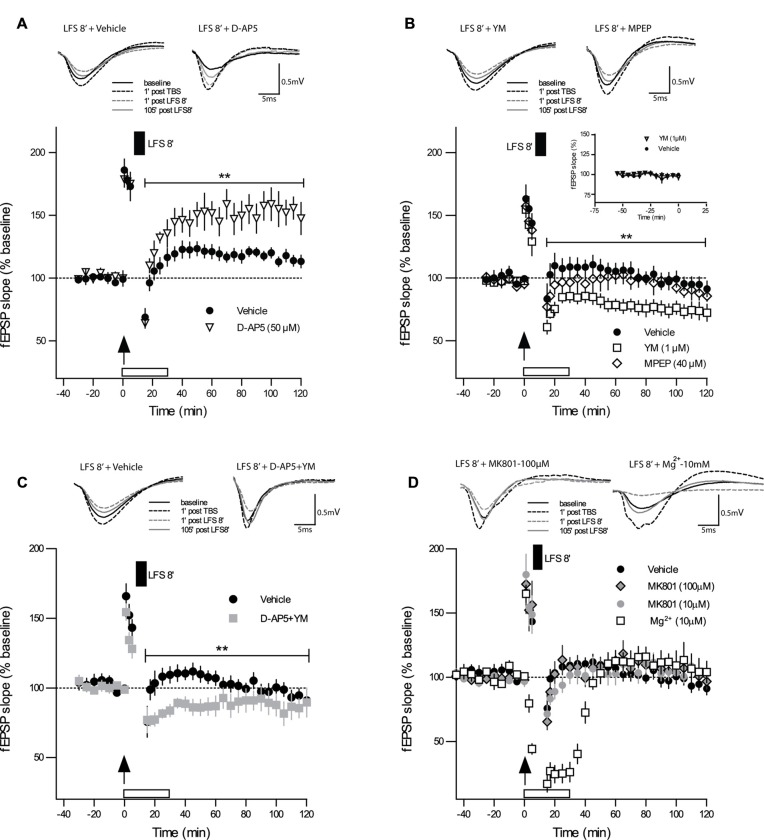
**Strong DP induced by DP8 is blocked by inhibition of NMDAR and facilitated by inhibition of mGluR1. (A)** The competitive NMDAR antagonist D-AP5 (50 μM; *n* = 6) caused a significant reduction in the magnitude of DP when compared to vehicle experiments (DP8 only; *n* = 8). Error bars represent SEM, *p* = 0.009. **(B)** While bath application of the selective mGluR5 antagonist MPEP (40 μM; *n* = 7) had no significant effect on DP, the mGluR1 inhibitor YM 298198 (1 μM; *n* = 7) caused a significant enhancement in the magnitude of LTP reversal compared to the vehicle group (DP8 only; *n* = 7). Error bars represent SEM, *p* = 0.009. Inset graph shows a series of extended baseline recordings comparing group of slices with YM 298198 application (*n* = 6) and vehicle application (*n* = 7). As indicated by almost identical curves, YM 298198 had no effect on basal synaptic transmission. DP8′ application is indicated by the filled box above the plot; arrow indicates time of TBS application. Traces show representative examples of fEPSPs recorded during baseline (black line), 1 min post-TBS induction (black broken line), 1 min post-LFS (gray broken line) and 120 min post-TBS (gray line). Calibration bars: 0.5 mV and 5 ms. See Figure 1 for further explanation. **(C)** The combined application of D-AP5 and YM 298198 resulted in a significant enhancement of DP *(p* < 0.010 RM-ANOVA, *n* = 6; control *n* = 9) which is, however, very similar to the effects of applying YM 298198 alone (shown in panel **B**; main group effect from 15 min to 120 min post LFS induction: *p* = 0.2406, RM-ANOVA); same data for vehicle (DP8) as in Figure 1C. **(D)** Bath-application of MK-801 (10 μM, *n* = 7 and 100 μM, *n* = 6) or high Mg^2+^ (10 mM, *n* = 6) did not inhibit DP and resulted in values at the level of controls (*n* = 9). These findings support a metabotropic operation of NMDAR during DP8′. **p ≤ 0.01.

We tested next whether the induction of DP by DP8 is dependent on the activation of NMDARs. When the competitive NMDAR antagonist D-AP5 (50 μM) was applied to slices from adult mice immediately after TBS, the initial potentiation did not differ from the vehicle control group 5 min post-TBS (Figure 2A; TBS + D-AP5: 175 ± 5%, *n* = 6; control TBS: 173 ± 11%, *n* = 10), indicating that the initial expression of LTP was not compromised by the post-TBS application of D-AP5. In contrast, the magnitude of DP in the group of slices treated with D-AP5 was significantly reduced for the entire recording interval, when compared to the vehicle group (Figure 2A; DP8 + D-AP5: 15 min 64 ± 3%, 120 min 147 ± 12%, *n* = 6; control DP8: 15 min 68 ± 6%, 120 min 113 ± 5%, *n* = 8; main effect of group: *F*_(1,12)_ = 9.97, *p* = 0.009; no significant interaction group × time, two-way RM-ANOVA on entire recording interval post-LFS). These findings indicate that DP induced by DP8 is NMDAR-dependent.

To test whether this strong form of DP is also mGluR-dependent, as suggested for DP induced by other protocols (Bashir and Collingridge, 1994; Bortolotto et al., 1994), we first bath-applied the selective mGluR5 antagonist MPEP (40 μM), which did not alter DP induced by DP8, excluding a role of mGluR5. The level of potentiation that remained after DP in MPEP-treated slices was not significantly different from those in the vehicle control (Figure 2B; DP8 + MPEP: 15 min 86 ± 5%; 120 min 86 ± 4%, *n* = 7; control DP8: 15 min 103 ± 9; 120 min 91 ± 6%, *n* = 7; *F*_(1,12)_ = 1.02, *p* = 0.333, RM-ANOVA). The selective mGluR1 antagonist YM 298198 (1 μM), in contrast, caused a marked enhancement of DP (see Figure 2B; DP8 + YM: 15 min 72 ± 4%; 120 min 72 ± 8%, *n* = 7; control DP8: 15 min 103 ± 9%, 120 min 91 ± 6%, *n* = 7; *F*_(1,12)_ = 9.761, *p* = 0.009, RM-ANOVA). To rule out a putative effect of YM 298198 on basal synaptic transmission we added a series of extended baseline recordings with this compound. No significant statistical differences were observed, as can be seen in the inset of Figure 2B. Hence, our results point to an involvement of NMDAR and mGluR1 but not mGluR5 in DP.

These results raised the question of whether NMDAR and mGluR1 work independently from each other or whether they are functionally linked. To approach this, we applied both inhibitors together to the same slice preparation and obtained a marked enhancement of DP (DP8 + D-AP5 + YM: 15 min 59 ± 15; 120 min 87 ± 11, *n* = 6; control DP8: 15 min 75 ± 11; 120 min 91 ± 4, *n* = 9; main effect of group for 2 h post induction: *F*_(1,17)_ = 8.329, *p* < 0.0103, RM-ANOVA, Figure 2C) which resembled the effect of YM when applied alone (main effect of group for 2 h post induction: *F*_(1,13)_ = 1.512, *p* = 0.2406, RM-ANOVA, Figures 2B,C).

### The Effect of DP8′ Includes a Metabotropic Function of NMDARs

The above experiments with the competitive NMDAR antagonist D-AP5 demonstrated the involvement NMDAR activation in DP8′ induction. D-AP5 blocks glutamate binding to the ligand-binding domain of the GluN2 subunit (Jespersen et al., 2014). Since a metabotropic mode of NMDAR operation has recently been reported to be important for the induction of LTD but not LTP (Nabavi et al., 2013; Aow et al., 2015; Dore et al., 2015; Weilinger et al., 2016), we wondered whether the induction of DP may also include metabotropic NMDAR actions. To approach this question, we applied the use-dependent NMDA-receptor antagonist MK-801 (10 μM) during DP8′. MK-801 is an irreversible open-pore blocker with an inhibition rate that is proportional to the level of channel activity. In these experiments we could not find a significant effect of MK-801 on the induction and maintenance of DP (Figure 2D; DP8 + MK-801: 15 min 71 ± 6, 120 min 102 ± 7, *n* = 7; control DP8: 15 min 75 ± 11; 120 min 91 ± 4, *n* = 9). Next, we used a ten-fold higher concentration of MK-801 (100 μM) and obtained very similar results (DP8 + MK-801: 15 min 65 ± 6, 120 min 96 ± 6, *n* = 6). To further address this question by an independent approach, we increased the Mg^2+^ concentration in ACSF immediately after the induction of LTP from 2.0 mM to 10 mM. Both, MK-801 and Mg^2+^ bind to sites inside the NMDAR channel pore (Huettner and Bean, 1988; Collingridge and Lester, 1989). As shown in Figure 2D, application of DP8 in high Mg^2+^ACSF resulted in a more pronounced decline of recordings as compared to MK-801 but the fEPSP-slope returned to a very similar level as obtained with MK-801 and in control experiments. Thus, different from the effects of D-AP5, application of MK-801 and high Mg^2+^ respectively, did not affect the induction of DP, supporting the possible participation of metabotropic NMDAR mechanisms in this process.

### LFS Delivered to Naive Slices Does Not Induce Permanent Changes in Synaptic Transmission nor Modulates LTP Induced by TBS

**Figure 3:**
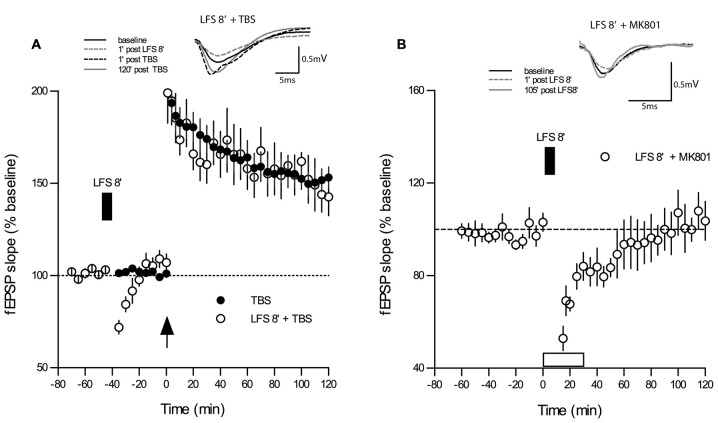
**LFS applied for 8 min at 5 Hz (DP8) to “naive” slices has no effect on subsequent LTP-induction. (A)** The magnitude and maintenance of potentiation in slices that underwent DP8′ 20 min before application of TBS (open circles; *n* = 8) are virtually the same as in “naïve” control slices (*n* = 10) without DP8 application. DP8 causes only an ephemeral depression for about 10 min (*p* < 0.05, Wilcoxon matched-pairs signed-rank test). LFS application is indicated by the filled box above the plot. Traces show typical examples of fEPSPs recorded during baseline (black line), 1 min post-LFS (gray broken line), 1 min post-TBS induction (black broken line), and 120 min post-TBS (gray line). Calibration bars: 0.5 mV and 5 ms. **(B)** Application of MK801 during the application of 5 Hz DP8′ extended the period of significant depression to about 35 min (*p* < 0.05, Wilcoxon matched-pairs signed-rank test, *n* = 6).

One reason for contradictory findings between studies on DP might lie in the fact that some of the frequencies used for DP induction may have more “plastic” effects on synapses than others, for example, a priming effect on subsequent LTP may occur as noted in several electrophysiological protocols (Albensi et al., 2007). To control for this possibility, we applied the same 5 Hz 8 min stimulus used in the DP8 protocol to *naive* slices. As shown in Figure 3, the application of 5 Hz for 8 min during baseline recordings (*n* = 8) caused only a short-lasting depression for approximately 10 min when compared to baseline recordings (*p* < 0.05, Wilcoxon matched-pairs signed-rank test, *n* = 10). Importantly, application of 1×TBS to the same population of synapses 20 min after recovery elicited an LTP which did not differ significantly from potentiation of the control LTP group (main effect of group: *p* = 0.434; no interaction group × time, two-way RM-ANOVA post-TBS).

Finally, we tested whether application of MK801 would affect the short-lasting depression of basal synaptic transmission during application of 5 Hz DP8′ and we found that this treatment extended the period of significant depression to about 35 min (*p* < 0.05, Wilcoxon matched-pairs signed-rank test, *n* = 6, Figure 3B).

## Discussion

Although DP was discovered more than 30 years ago (Hesse and Teyler, 1976; Barrionuevo et al., 1980), its mechanisms and physiological functions are still far from being completely understood. In the present study, we investigated the role of group I mGluR subtypes in the succession of two different types of synaptic plasticity, i.e., the LTP induction at naive Schaffer collateral-CA1 synapses followed 6 min later by LTP reversal triggered by the application of LFS of 5 Hz for either 2 or 8 min (DP2 and DP8, respectively). As pharmacological tools we chose the high-affinity, selective, and non-competitive mGluR1 antagonist YM298198 (YM, 6-Amino-*N*-cyclohexyl-3-methylthiazolo[3,2-*a*]benzimidazole-2-carboxamide hydrochloride, IC50 = 24 nM; Kohara et al., 2005) and the highly selective, non-competitive antagonist at the mGlu5 receptor subtype MPEP hydrochloride, IC50 = 36 nM (Gasparini et al., 1999), respectively. Binding of YM-298198 to mGluR5 is more than 100-fold less potent compared to mGlu1 and no activity of YM was found at any other metabotropic or ionotropic glutamate receptor up to a 400-fold concentration of its IC50 (Kohara et al., 2005). The mGluR5 antagonist MPEP, on the other hand, is highly selective at the human mGlu5a receptor expressed in recombinant cells, but does not show agonist or antagonist activities at cells expressing the human mGlu1b receptor at concentrations up to 30 mM (Gasparini et al., 1999). Furthermore, MPEP has no agonist or antagonist activity at any other metabotropic or ionotropic glutamate receptor at concentrations of 10 mM (Gasparini et al., 1999).

In this study, we found an impairment of TBS-induced LTP *in vitro* by bath-application of MPEP while YM had no effect under the same conditions. Since application of the competitive NMDAR antagonist D-AP5 deteriorated TBS-LTP as well, our experiments underline that LTP in area CA1 in mice does not only require the activation of NMDAR, but also concomitant activation of mGluR5. This is particularly interesting in light of earlier findings that activation of group I mGluRs in hippocampal neurons leads to excitatory effects and potentiation of NMDA-mediated responses (Conn and Pin, 1997) and that mGluR5 and NMDA work in tandem to regulate synaptic strength and flexibility (De Blasi et al., 2001). The inhibition of TBS-LTP by MPEP found in our study corroborates previous reports showing that inhibition of mGluR5 results in an impairment of hippocampal LTP in rats *in vivo* and *in vitro* (Manahan-Vaughan, 1997; Balschun and Wetzel, 2002; Naie and Manahan-Vaughan, 2004; Manahan-Vaughan and Braunewell, 2005; Nagaraja et al., 2005; Kulla and Manahan-Vaughan, 2008), and that mGluR5 knock-out mice have a deficit in CA1-LTP (Lu et al., 1997).

The lack of any effect of YM application on LTP is in apparent contradiction to data from mGluR1 knock-out mice which displayed impaired CA1-LTP (Aiba et al., 1994) and to studies that used group I mGluR inhibitors with a stronger affinity to mGluR1 than mGluR5 (like 4-CPG ((S)-4-carboxyphenylglycine; Brabet et al., 1995)) leading to a deficit in CA1-LTP in rats (Wilsch et al., 1998; Balschun et al., 1999a). These differences in the recruitment of group I mGluR subtypes are most likely due to species and strain differences in the physiological circuits engaged in the activation of mGluR1 and mGluR5.

Group I mGluRs appear to be of particular importance for hippocampal function and memory. Their role in different forms of synaptic plasticity such as LTP and LTD has been extensively studied (Bortolotto et al., 1994; Wilsch et al., 1998; Balschun et al., 1999a; Raymond and Redman, 2002; Zho et al., 2002; Manahan-Vaughan and Braunewell, 2005; Neyman and Manahan-Vaughan, 2008) (see Anwyl, 2009; Lüscher and Huber, 2010 for further references). However, none of these pharmacological studies clearly elucidated which group I mGluR subtype was responsible for the observed effects. Here, we found that the inactivation of mGluR5 with MPEP, but not the inhibition of mGluR1 with YM298198, blocked the induction and maintenance of LTP. In contrast to the involvement of mGluR5 in LTP induction, this subtype does not seem to have a role in LTP reversal. Instead, long-lasting DP (as induced by the DP8 protocol) turned out to be NMDA-dependent and tonically suppressed by mGluR1 because inactivation of this mGluR subtype enhanced the magnitude of DP. This is the first *in vitro* demonstration that time-dependent DP is concomitantly regulated by NMDARs and mGluRs, in contrast to earlier *in vitro* studies suggesting that this form of synaptic plasticity relied exclusively either on NMDARs or group I mGluRs in CA1 (reviewed in Sanderson, 2012; see also “Introduction” Section).

The stimulation paradigms we employed to induce LTP and DP are based on the “hippocampal-theta-rhythm”, a large amplitude oscillation seen in electroencephalographic recordings in the range of 4–8 Hz (Vanderwolf, 1969; Buzsáki et al., 1983), and as such, may be considered as better approximating the *in vivo* physiological functioning than HFS and other types of LFS. The DP8 protocol resulted in a robust and reproducible reset of potentiation surpassing DP-inducing protocols of 2, 3 and 5 min LFS, which were “duration-dependently” less effective. Noteworthy, the non-saturated level of DP that was achieved with DP8 is reminiscent of previous *in vivo* or *in vitro* studies that likewise employed 5 Hz LFS to induce DP (Fujii et al., 1991; O’Dell and Kandel, 1994; Qi et al., 2013).

The requirement for NMDARs stimulation during theta-induced DP was already established in young rats *in vitro* (O’Dell and Kandel, 1994) as well as *in vivo* (Barr et al., 1995). Milner et al. (2004) replicated this requirement in mice of similar age as we used for our studies, but they employed LFS at 1 Hz to induce DP. Thus, our results with the competitive NMDAR antagonist D-AP5 extend the above, by showing for the first time that in slices from adult mice DP induced by a combination of theta-based stimuli also demands NMDAR activation.

Recently, a number of studies added a new facet to the known mechanisms of NMDARs. Nabavi et al. (2013) reported that an LTD induced by LFS of 900 pulses at 1 Hz was prevented by the widely-used competitive NMDAR antagonist D-AP5 that blocks glutamate binding to the ligand-binding domain of the GluN2 subunit (Jespersen et al., 2014), but not by the irreversible use-dependent open-pore blocker MK-801 (100 μM; Huettner and Bean, 1988) or 7-chlorokynurenate (7CK), an antagonist at the glycine-binding site of the GluN1 subunit (Kemp et al., 1988). The study indicated that ligand binding to NMDARs is sufficient to induce LTD but neither ion flow through NMDARs nor a rise of intracellular Ca^2+^ is required. However, core findings of this study could not be reproduced by others. Babiec et al. (2014) reported an inhibition of electrically and chemically induced types of NMDAR-dependent LTD by even tenfold lower concentrations of MK-801 (10 μM) and a dependency of LTD induction on intracellular Ca^2+^. Volianskis et al. (2015) re-investigated the effects of antagonizing the glycine-binding site of the GluN1 subunit with the potent and highly specific glycine site antagonist L-689,560 (Grimwood et al., 1995) and obtained a complete block of the same type of LTD as employed by Nabavi et al. (2013). Thus, although there is growing evidence of metabotropic actions of NMDAR (Aow et al., 2015; Dore et al., 2015, 2016; Weilinger et al., 2016) being involved in functions like the inhibition of excitatory synaptic transmission by amyloid-ß (Aß) peptides (Kessels et al., 2013), NMDAR trafficking (Dore et al., 2016) and structural plasticity (Stein et al., 2015), the involvement of metabotropic NMDAR actions in LTD induction remains controversial. Given that LTD and DP share several mechanisms (Huang et al., 2001) it was tempting to test whether NMDAR-dependent DP8′ also depends on metabotropic NMDAR-function. Our findings are in support of a metabotropic function of NMDARs during DP induction. MK-801 applied at concentrations of either 10 μM or 100 μM did not block DP8′ while a clear inhibition was obtained with 50 μM D-AP5. However, a stringent methodical limitation of the study is the fact that there was only a narrow time-window of 6 min after TP-induction to bath-apply the compounds before DP-induction. For this reason, we also tested the effect of increasing the concentration in the bath-solution from 2 mM to 10 mM immediately after LTP-induction, a procedure that leads to a rapid blockade of the channel pore (Collingridge and Lester, 1989). Given that this procedure also failed to block DP it appears reasonable to conclude that metabotropic NMDAR functions are involved in the induction of DP.

Interestingly, application of 10 μM MK-801 delayed the return of the short-lasting depression when DP8′ was applied to naive synapses. This finding is reminiscent of data that demonstrate that the decay of potentiation is dependent on the recording frequency and NMDAR activation (Volianskis and Jensen, 2003). However, in our case of an LFS-induced depression, interference with NMDAR function appeared to delay the return to baseline.

Regarding the function of group I mGluRs in DP, the group I mGluR agonist DHPG has been reported to induce reliably DP at hippocampal CA1 synapses in rats (Zho et al., 2002). This phenomenon is partially mediated by rapid AMPA receptors internalization from the post-synaptic membrane and mechanistically different from NMDAR-dependent DP (see Huang et al., 2001; Sanderson, 2012). The chemical induction of DP by DHPG, which is at least partially mediated by mGluR5 activation (Zho et al., 2002), raises the question of whether mGluR5 activation is required for DP induction by *electrical* stimulation in the mouse HC. Our experiments in this study uncover a clear-cut mechanistic difference between chemical and electrical induction of DP because the reversal of LTP induced by DP8 turned out to be independent of mGluR5 activation but facilitated by inhibition of mGluR1.

Interestingly, mGluR1 seems to control or override the involvement of NMDAR in DP because in the current study, YM and D-AP5 applied together yielded almost the same strengthening of DP as YM alone. Hsu et al. (2011) suggested recently that “CA1 synaptic plasticity is regulated by the result of competition between NMDARs and mGluR5 receptors”. Although a similar competition is likely to occur also between NMDARs and mGluR1, an interaction between mGluR1 and metabotropic NMDAR actions cannot be simply explained by data from published studies because they are exclusively based upon the use of the competitive NMDAR antagonist D-AP5 which does not allow to distinguish between ionotropic and metabotropic NMDAR actions. Although there are not yet any other studies that point to a particular mechanism that may underlie this mGluR1-mediated control, it seems to be likely that mGluR1 acts upstream of NMDAR. One putative pathway could be the activation of phospholipase C (PLC) by mGluR1, which results in the hydrolysis of PIP2 to IP3 and DAG, the latter of which activates PKC. PKC, in turn, may activate members of the Src family of non-receptor tyrosine kinases (SFKs) such as Scr and Fyn. SFKs closely associate via indirect and direct binding mechanisms with NMDAR (Vissel et al., 2001; Groveman et al., 2012) described a downregulation of recombinant NR1/2A receptors by tyrosine dephosphorylation that required agonist binding, but no ion flux. The study provides evidence for Src-mediated phosphorylation of a ring of tyrosines on the NMDAR itself as being important for the metabotropic NMDAR function. Thus, an equilibrium between phosphorylation/dephosphorylation of certain tyrosine residues at NMDARs could provide the trigger for a conformational change in the NMDAR cytoplasmic domain (Aow et al., 2015) enabling its metabotropic mode of operation.

Another plasticity-relevant pathway that has been implicated in metabotropic NMDAR actions is the activation of p38 MAPK (Nabavi et al., 2013; Birnbaum et al., 2015) which has been shown to be central to DP (Liang et al., 2008) and AMPAR endocytosis (Huang et al., 2004) However, the two pathways mentioned above are just two options how mGluR1 and metabotropic NMDAR actions could control the expression of DP. Future studies are required to identify the precise signaling mechanisms.

Our experiments support previous findings of Huang et al. (2001) that activation of mGluRs is not an absolute requirement for DP to occur. They reported that application of the selective group I mGluR antagonist 1-Aminoindan-1, 5-dicarboxylic acid (AIDA, 500 μM) and the non-selective group I/II mGluR antagonist (S)-α-Methyl-4-carboxyphenylglycine (MCPG; 500 μM) did not significantly affect the induction of DP by LFS at 2 Hz for 10 min. By using a protocol that has virtually no effects on naive synapses, our results demonstrate for the first time a specific role of each group I mGluR subtype in the succession of LTP to DP. They reveal an inhibitory role of mGluR1 in electrically-induced DP in hippocampal slices, providing further evidence that mGluR1 has multiple functions in learning-related plasticity. Moreover, these results make mGluR1 a good candidate to study the putative role of DP in certain HC-dependent memory behavioral tasks. As previously reported, mGluR1 activity has a potential role for DP at thalamic input synapses onto the lateral amygdale that underlie fear extinction (Kim et al., 2007) To this end, the ability of group I mGluR inhibition to impair learning (Aiba et al., 1994; Rodrigues et al., 2002; Kulla and Manahan-Vaughan, 2008) might be due to an impairment of LTP (mGluR5) and/or augmented DP, depending on the particular learning protocol. Note that Qi et al. (2013) reported that DP induced by novelty exploration is mGluR5-dependent, which is in apparent contradiction to the findings in the present work. Thus, the involvement of mGluR5 in certain types of synaptic plasticity seems to depend on the particular experimental conditions and the history of activation. In agreement with the latter, mGluR5 was demonstrated to be important for setting the “molecular switch”, a mechanism that regulates the need for the synaptic activation of mGluRs during the induction of LTP (Bortolotto et al., 2005). It is likely that the use of different experimental conditions for LTP and DP results in the activation of different biochemical processes that lead to different synaptic effects (Masino and Dunwiddie, 1999). In this respect, and according to the Bienenstock-Cooper-Munro (BCM) theory (Bienenstock et al., 1982; Cooper and Bear, 2012), the use of the “plasticity-neutral” theta frequency of 5 Hz is an additional advantage of our protocol as compared to the use of other frequencies to induce DP (Huang et al., 2014). This is confirmed by our data showing that LFS-5 Hz does not change the magnitude of the following 1×TBS induction of LTP for at least 2 h. This is in agreement with an earlier study which reported that delivery of single pulses at 5 Hz to naive synapses does not cause any persistent change in baseline synaptic transmission (Stäubli and Chun, 1996).

## Conclusion

The present study provides several converging lines of evidence that mGluR5 and mGluR1 subtypes are reciprocally involved in 1×TBS-LTP and DP. mGluR5 is crucial for proper induction of 1×TBS-LTP and does not affect DP. In contrast, mGluR1 tonically suppresses DP but has no role in 1×TBS-LTP. Both types of synaptic plasticity evaluated herein are NMDAR-dependent because inhibition of these receptors prevents 1×TBS-LTP and DP. Moreover, the involvement of NMDARs in DP appears to include metabotropic functions that are independent of any ion flux through the channel pore.

Although not much is known about the physiological and pathophysiological importance of DP, it might serve as a mechanism required for cognitive flexibility, i.e., the ability to properly change behavior upon environmental demands (Kulla and Manahan-Vaughan, 2008; Zhang et al., 2011; Hampshire et al., 2012). Moreover, despite receiving less attention than its “sister-process” LTD, DP is possibly similarly ubiquitous as LTP (Villarreal et al., 2002). Since reduced cognitive flexibility is a characteristic trait of patients suffering from neurodegenerative and neuropsychiatric disorders such as Alzheimer’s disease, Fronto-temporal dementia and Fragile × Syndrome, the elucidation of the cellular and molecular mechanisms of DP has an important pathophysiological and therapeutical dimension.

## Author Contributions

AL-H, EF and TA carried out experimental work and analyses and contributed equally to the work as first author. AL-H and DB wrote the article.

## Conflict of Interest Statement

The authors declare that the research was conducted in the absence of any commercial or financial relationships that could be construed as a potential conflict of interest. The reviewer GRJG and handling Editor declared their shared affiliation, and the handling Editor states that the process nevertheless met the standards of a fair and objective review.
